# An enzyme-responsive metal-enhanced near-infrared fluorescence sensor based on functionalized gold nanoparticles†
†Electronic supplementary information (ESI) available: Detailed information on the synthetic routes to various ligands, characterization data, additional figures, schemes, tables and general experiment procedures are provided. See DOI: 10.1039/c5sc01850a



**DOI:** 10.1039/c5sc01850a

**Published:** 2015-06-19

**Authors:** Zhanghua Zeng, Shin Mizukami, Katsumasa Fujita, Kazuya Kikuchi

**Affiliations:** a Division of Advanced Science and Biotechnology , Osaka University , Osaka , 565-0871 , Japan . Email: kkikuchi@mls.eng.osaka-u.ac.jp; b Department of Applied Physics , Graduate School of Engineering , Osaka University , Osaka , 565-0871 , Japan; c Immunology Frontier Research Centre , Osaka University , Osaka , 565-0871 , Japan

## Abstract

Near-infrared (NIR) fluorescence imaging is promising due to the high penetration depths and minimal levels of autofluorescence in living systems. However, it suffers from low fluorescent quantum yield, and metal-enhanced fluorescence (MEF) is considered to be a promising technique to overcome this. Stimuli-responsive NIR fluorescence enhancement shows remarkable potential for applications in medical imaging and diagnosis. Herein, we successfully fabricated an enzyme-responsive near-infrared sensor based on MEF by functionalizing gold nanoparticles with NIR fluorophores and enzyme-responsive self-aggregation moieties. The NIR fluorescence of fluorophores on the gold nanoparticles was significantly enhanced due to increases both in the light scattering intensity and in the radiative decay rate (*k*_r_) of the NIR fluorophores, along with relatively small variation in the nonradiative decay rate. This novel strategy for NIR fluorescent sensors should be particularly promising for NIR fluorescence imaging of enzyme activities and early diagnosis based on rationally designed nanomaterials.

## Abstract 2

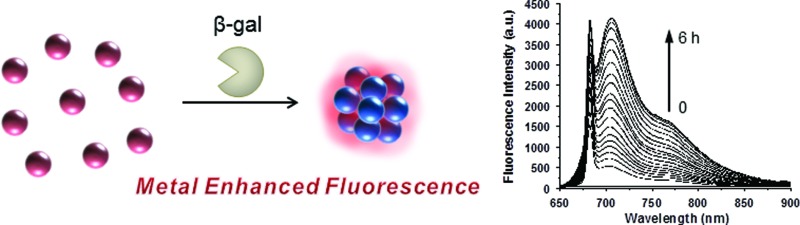
An enzyme-responsive NIR nanosystem based on MEF was fabricated by surface functionalization of gold nanoparticles. Sensors based on this strategy are promising for enzyme detection in early diagnostic imaging and *in vivo* applications.

## Introduction

Near-infrared (NIR) fluorescence imaging continues to attract attention because, in the physiologically relevant optical window (700–900 nm), the features of this technique include minimal levels of interfering absorption from biological samples, reduced scattering, enhanced tissue penetration depth, and minimal levels of autofluorescence.^1–6^ However, most NIR fluorophores exhibit fluorescence quantum yields (*Φ*_F_) much lower than those of visible-wavelength fluorophores. For instance, indocyanine green (ICG), one of the most commonly used dyes in medical diagnostics, has a low *Φ*_F_ of 0.012 in aqueous media,^7^ whereas fluorescein, a typical green fluorescent dye, has a *Φ*_F_ of 0.91.^8^ The low *Φ*_F_ of NIR fluorophores significantly limits their applications in medical imaging. Moreover, the development of NIR fluorescent sensors based on fluorescence resonance energy transfer or photo-induced electron transfer is more problematic and less efficient than for visible-wavelength fluorophores. Therefore, the development of a general approach for NIR fluorescence enhancement has become highly important and urgent.

Recently, many reports have shown that localized surface plasmon resonance (LSPR) of noble metals can enhance the fluorescence of proximal fluorophores when the architectures of metallic nanostructures and fluorophores are properly engineered.^9–21^ This phenomenon is named as metal-enhanced fluorescence (MEF).^22^ Plenty of surface-immobilized metallic nanostructures for MEF have been developed and the advantages of MEF include the increased photostability of fluorophores,^23^ and improved detection of protein^24^ and DNA/RNA.^25^ Although the mechanism of MEF is not fully understood, it is indicated that the strong local electric field of LSPR causes an increase in the radiative decay rate (*k*_r_) of fluorophores near the metallic nanostructure.^12,26^ On the other hand, it is well known that noble metal nanostructures such as gold nanoparticles (AuNPs) strongly quench the fluorescence of neighboring fluorophores. When the distance between the gold surface and the fluorophore is very short (<5 nm), or when the electromagnetic resonance coupling between the LSPR and the fluorophore emission is very small, fluorescence quenching is observed due to the predominance of the nonradiative decay rate (*k*_nr_).^27–31^ In principle, the change from fluorescence enhancement to fluorescence quenching depends on the relative magnitudes of *k*_r_ and *k*_nr_ of the fluorophore. Thus, the fluorescence enhancement caused by AuNPs is a function of both the proximity and the electromagnetic resonance coupling of the fluorophores and AuNPs. So far, most studies on MEF have focused on the fabrication of systems that allow for a more selective control of the distance between the fluorophore and the gold film surface, and of the electromagnetic resonance coupling.^32–38^ Nevertheless, stimuli-responsive enhancement of NIR fluorescence using noble metal LSPR, especially that based on the tuning of LSPR, has hardly been developed, despite the fact that it shows more potential for significant imaging applications in medicine.

Thus, we planned to develop MEF-based NIR fluorescent sensors by adequately tuning the distance between the metal surface and fluorophores in response to analytes. Recently, we reported that β-galactosidase (β-gal) can shift the LSPR absorption spectrum of AuNPs functionalized with specific β-gal substrate ligands from the visible to the NIR region.^39^ This result prompted us to exploit enzyme-triggered LSPR changes to obtain MEF. Here we report a β-gal-responsive metal-enhanced NIR fluorescence sensor based on functionalized AuNPs.

## Results and discussion

**Scheme 1:**
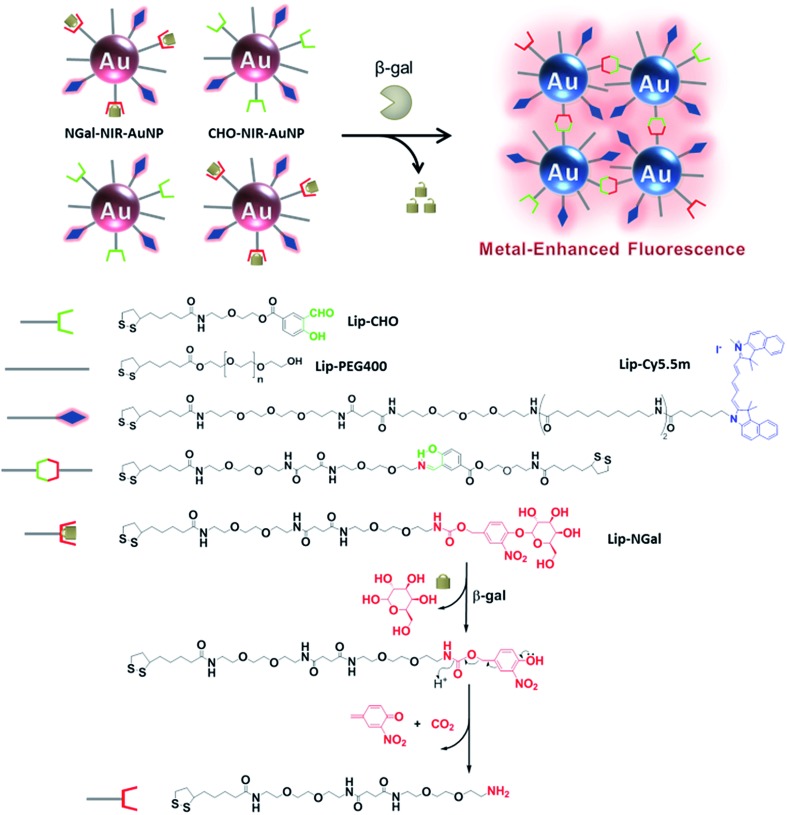
Schematic illustration of the proposed mechanism of the enzyme-responsive fluorescence enhancement by NGal-NIR-AuNPs/CHO-NIR-AuNPs.

The mechanism of the enzyme-responsive NIR fluorescence enhancement is illustrated in Scheme 1. The prepared nanoparticles, NGal-NIR-AuNPs and CHO-NIR-AuNPs, were anchored with β-gal-substrate ligands (Lip-NGal) and with aldehyde ligands (Lip-CHO), respectively. NIR-fluorophore ligands (Lip-Cy5.5m) and polyethylene glycol ligands (Lip-PEG_400_) as co-ligands for stabilization in solution were attached to both types of AuNPs. The synthetic routes to the ligands Lip-NGal, Lip-CHO, and Lip-Cy5.5m are summarized in Schemes S1–S3†. Details of the synthetic procedures, the characterization of the intermediates and ligands, the synthesis of gold nanoparticles, and the fabrication of functionalized gold nanoparticles (NGal-NIR-AuNPs and CHO-NIR-AuNPs) are also provided in the ESI.†

The ratio of the attached ligands on the nanoparticle surface was determined by the dye molar extinction coefficient and HPLC analysis (Scheme S4†). As a result, the ratio of Lip-NGal (or Lip-CHO), Lip-Cy5.5m, and Lip-PEG_400_ was 1/0.12/1.26 and 1/0.10/1.19 for the NGal-NIR-AuNPs and CHO-NIR-AuNPs, respectively. The results show slight differences from those of the modification-reaction solutions (Lip-NGal (or Lip-CHO) : Lip-Cy5.5m : Lip-PEG_400_ = 1/0.2/1), presumably due to the nature of the ligand pendants (hydrophilic sugar *versus* hydrophobic dye), lengths of the linkers, and the overall sizes of the ligands.^40–42^ The fluorescence of the surface-attached Cy5.5m was expected to be quenched by AuNPs due to the negligibly small overlap between the LSPR absorption (∼520 nm) and the emission (∼707 nm) spectra (Fig. S1†). After the enzymatic substrate hydrolysis of NGal-NIR-AuNPs, a self-immolative elimination reaction *via* the formation of a quinone methide occurs and the subsequent decarboxylation rapidly produces a primary amine that reacts with an aldehyde on a neighboring CHO-NIR-AuNP to form a Schiff base. As a result, the inter-nanoparticle distance decreases and the LSPR shifts towards the NIR region, as Mirkin *et al.* reported.^43^ Such an increase in the spectral overlap between LSPR and emission enhances the electromagnetic resonance coupling, and the fluorescence is enhanced.

**Fig. 1:**
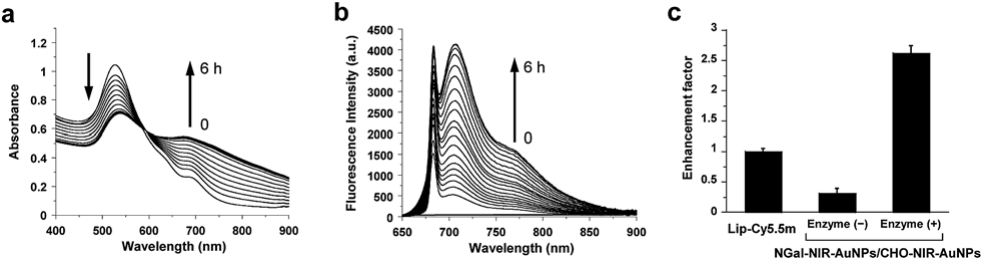
(a) Time-dependent absorbance spectral changes of NGal-NIR-AuNPs/CHO-NIR-AuNPs (2.0 nM) in the presence of β-gal (1.0 μM). (b) Time-dependent fluorescence spectral changes (*λ*_ex_ = 680 nm) of NGal-NIR-AuNPs/CHO-NIR-AuNPs (1.0 nM) in the presence of β-gal (1.0 μM). (c) Relative fluorescence intensity in the absence or presence of β-gal, where the enhancement factor of Lip-Cy5.5m was normalized to 1. All of the samples were suspended in PBS (pH 7.4) at 37 °C.

To verify the β-gal-induced LSPR absorption spectral changes of the mixture of the two different gold nanoparticles (NGal-NIR-AuNPs/CHO-NIR-AuNPs), the evolution of the visible-NIR absorbance spectrum of NGal-NIR-AuNPs/CHO-NIR-AuNPs was measured over time (Fig. 1a). The spectra show a typical LSPR peak at 525 nm, which is 5 nm red-shifted with respect to that of the citrate-capped gold nanoparticles due to the ligand-exchange effect,^44^ and they have a shoulder around 680 nm that arises from the absorbance of Cy5.5m in phosphate buffered saline (PBS) (Fig. S2†). As expected, the addition of β-gal causes a decrease in the intensity of the absorbance at 525 nm over time, along with a gradual increase in absorption near 700 nm due to the aggregation of gold nanoparticles. In comparison, the absorption spectrum of NGal-NIR-AuNPs does not change in the presence of β-gal (Fig. S3†). These results indicate that the formation of the Schiff base induces a shift of the LSPR absorption toward the NIR region.

The fluorescence of NGal-NIR-AuNPs/CHO-NIR-AuNPs in PBS at 37 °C was partially quenched relative to that of Lip-Cy5.5m, because the fluorophores are deposited on the Au surface *via* a moderately long linker (>9 nm). Upon the addition of β-gal, the fluorescence was enhanced in a time-dependent manner by a factor of 5.2 at 1 h, 6.7 at 2 h, and 7.8 at 6 h of incubation, respectively (Fig. 1b and S4†). These results indicate that the response time was relatively fast initially, and afterwards became slower. This could be satisfactory for in-demand imaging techniques, such as *in vitro* enzyme detection and early diagnosis. However, we realize the improvement of reaction kinetics is very important for versatile imaging applications as well as improvement of the enhancement factor. Relative to the fluorescence of free ligand Lip-Cy5.5m (at the same concentration) in PBS, the fluorescence was enhanced by a factor of 2.6 (Fig. 1c and Scheme S4†).

The excitation spectra were similarly enhanced (Fig. S5†). A prolonged incubation time did not afford significant further spectral changes. The fluorescence enhancement was also examined in a biological solution containing BSA (0.1%, w/w) and glycine (0.25%, w/w). Under these conditions, the enhancement factor slightly decreased to 4.6-fold at 1 h and 6.2-fold at 6 h, keeping around 90% and 81% enhancement, respectively, compared to that in PBS (Fig. S4†). The small amount of disruption indicates its practicability in biological media, although complete bioorthogonality is more promising.

Although various ratios of Lip-NGal (or Lip-CHO) and Lip-Cy5.5m (from 10 : 1 to 1 : 1 at the concentration ratio in the modification reaction) were employed to modulate the properties of the nanoparticles, variation of these ratios proved to have no significant effect on the enhancement factor (Fig. S6†). The fluorescence of NGal-NIR-AuNPs is hardly affected by the presence of β-gal (Fig. S7†). This strongly indicates that the enzyme-responsive fluorescence enhancement of NGal-NIR-AuNPs/CHO-NIR-AuNPs is induced by an increase in the spectral overlap between the LSPR of gold nanoparticles and the emission of the fluorophores, which causes the electromagnetic resonance coupling.

It was difficult to directly monitor the enzymatic hydrolysis and Schiff base formation on the AuNP surface. Alternatively, the enzymatic reaction of Lip-NGal was analyzed. The absorption spectra (Fig. S8†) and HPLC chart (Fig. S9†) show the reaction proceeded in a conversion yield of almost 100% within 10 min. The subsequent Schiff base formation with Lip-CHO proceeded relatively slowly, and the reaction yield was almost 50–60% after 4 h (Fig. S10†). These results suggest that the slow kinetics of the β-gal-triggered spectral changes of NIR-AuNPs (Fig. 1a and b) is mainly due to the slow rate of the Schiff base formation.

**Fig. 2:**
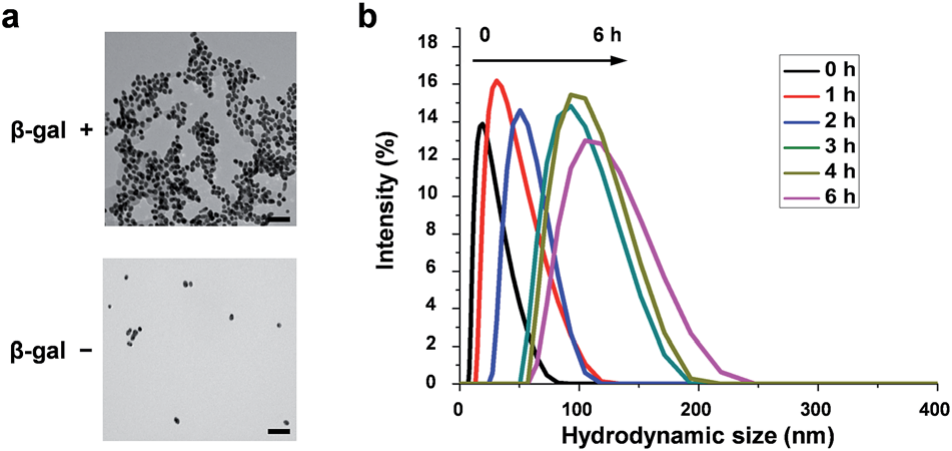
Morphological variation of functionalized gold nanoparticles, induced by enzymatic reaction in PBS at 37 °C. (a) TEM images of NGal-NIR-AuNPs/CHO-NIR-AuNPs (2.0 nM) in the absence (left) and presence (right) of β-gal (1.0 μM) after 5 h. Scale bars: 100 nm. (b) Time-dependent variation of the hydrodynamic diameter distributions of NGal-NIR-AuNPs/CHO-NIR-AuNPs (2.0 nM) measured by DLS after the addition of β-gal (1.0 μM).

The morphological variation was confirmed by transmission electron microscopy (TEM) (Fig. 2a). Without β-gal, NGal-NIR-AuNPs/CHO-NIR-AuNPs were quite well dispersed. Upon addition of β-gal, the nanoparticles aggregated. The hydrodynamic size of nanoparticles measured by dynamic light scattering (DLS) showed a gradual increase with β-gal from 25 nm to 137 nm (Fig. 2b). Time-dependent zeta-potential (*ζ*) measurements showed a drastic increase from –3.4 to +6.2 mV in 20 min and a subsequent slow decrease to +3.5 mV over time (Fig. S11†). All data indicate that enzymatic production of primary amines on the nanoparticle surface occurs in the initial stage (∼20 min) and the Schiff-base formation accompanied with the consumption of the amines progresses more slowly, in agreement with HPLC analysis (Fig. S9 and S10†).

Next, to validate the mechanism of enzyme-responsive NIR fluorescence enhancement, we synthesized hollow AuNPs (HGNs) with an LSPR peak at 700 nm (matching well with the emission of Cy5.5m),^45^ and modified them with Lip-Cy5.5m (Fig. S12†). The fluorescence of these fluorophore-modified Lip-Cy5.5m@HGNs was remarkably enhanced by a factor of 4.2 relative to that of Lip-Cy5.5m at the same concentration (Fig. S13†), indicating that the sufficient spectral overlap between LSPR and NIR emission efficiently induces the fluorescence enhancement.

**Fig. 3:**
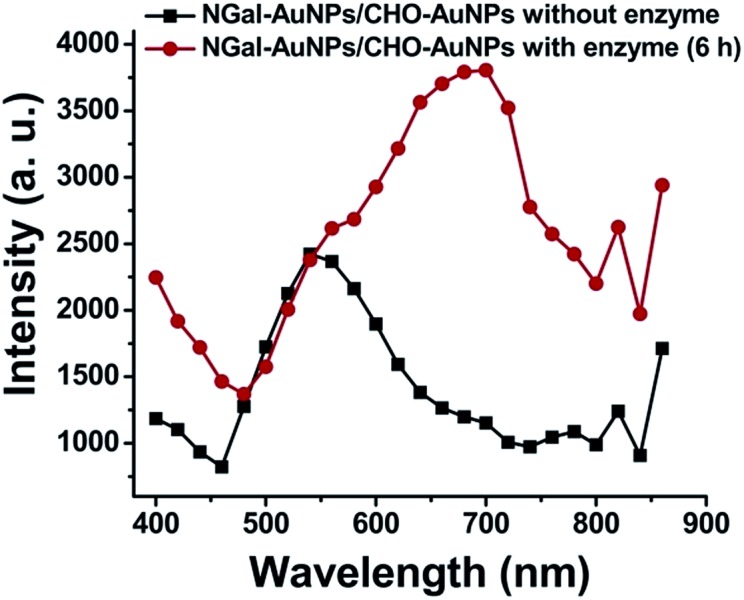
Light scattering spectral shift of NGal-AuNPs/CHO-AuNPs (1.0 nM) in the absence and presence of enzyme (6 h) in PBS (pH 7.4) at 37 °C.

Then, the essential mechanism of responsive MEF was investigated. The increase in scattering efficiency enhances the amount of light absorbed by the proximal fluorophores and contributes to the fluorescence enhancement.^12,14^ To evaluate the light scattering effect, fluorescent dye-free NGal-AuNPs/CHO-AuNPs were prepared on the assumption that they show the same light scattering intensity changes as the dye-containing NGal-NIR-AuNPs/CHO-NIR-AuNPs. In the presence of β-gal, the scattering intensity of NGal-AuNPs/CHO-AuNPs drastically increased in the NIR region (Fig. 3).

The effect of AuNPs aggregation on the fluorescence radiation process was also investigated. Fluorescence lifetime measurements revealed the average fluorescence lifetimes (*τ*_ave_) of Lip-Cy5.5m, and NGal-NIR-AuNPs/CHO-NIR-AuNPs in the absence and presence of β-gal were 835 ps, 274 ps and 202 ps, respectively (Table 1 and S2, and Fig. S14†). The *Φ*_F_ of Lip-Cy5.5m and NGal-NIR-AuNPs/CHO-NIR-AuNPs without and with enzyme were 0.18, 0.057, and 0.36, respectively (Table 1). From the *τ*_ave_ and *Φ*_F_, the kinetic parameters of the radiation process were calculated (Table 1). The nonradiative kinetic parameter *k*_nr_ of NGal-NIR-AuNPs/CHO-NIR-AuNPs without enzyme is 3.3 × 10^9^ s^–1^, which is a factor of 3.3 higher than that of Lip-Cy5.5m (1.0 × 10^9^ s^–1^). Concomitantly, the radiative kinetic parameter *k*_r_ is 2.1 × 10^8^ s^–1^, which is almost unchanged from that of Lip-Cy5.5m (2.2 × 10^8^ s^–1^). These results indicate that the AuNPs significantly quench the fluorescence of the surface-modified fluorophores and that the small enhancement of *k*_r_ is probably due to negligible coupling between LSPR and emission. Interestingly, *k*_r_ with enzyme is 1.8 × 10^9^ s^–1^, which is an enhancement by a factor of 8.2 and 8.6 relative to Lip-Cy5.5m and NGal-NIR-AuNPs/CHO-NIR-AuNPs without enzyme, respectively. On the other hand, *k*_nr_ is 3.2 × 10^9^ s^–1^, which is slightly lower than that without enzyme and about 3 times larger than that of Lip-Cy5.5m. Overall, NGal-NIR-AuNPs/CHO-NIR-AuNPs show an enhancement of the *Φ*_F_ by a factor of approximately 6 in response to β-gal, and it is mostly due to a significant increase in *k*_r_.

**Table 1:** Fluorescence quantum yield (*Φ*_F_), radiative (*k*_r_) and nonradiative (*k*_nr_) rate constants of Lip-Cy5.5m, NGal-NIR-AuNPs/CHO-NIR-AuNPs without and with enzyme, and Lip-Cy5.5m@HGNs

Samples	*Φ*_F_	*k*_r_ (s^–1^)	*k*_nr_ (s^–1^)
Lip-Cy5.5m	0.18	2.2 × 10^8^	1.0 × 10^9^
NGal-NIR-AuNPs/CHO-NIR-AuNPs without enzyme	0.057	2.1 × 10^8^	3.3 × 10^9^
NGal-NIR-AuNPs/CHO-NIR-AuNPs with enzyme (6 h)	0.36	1.8 × 10^9^	3.2 × 10^9^
Lip-Cy5.5m@HGNs	0.63	5.3 × 10^9^	3.1 × 10^9^

**Fig. 4:**
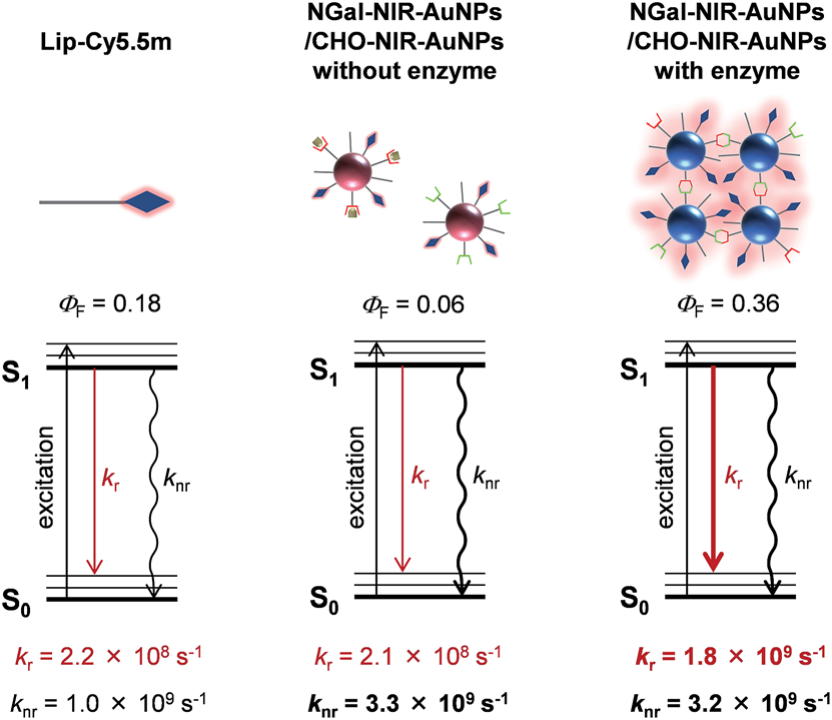
Plausible mechanism of the β-gal-responsive metal-enhanced NIR fluorescence enhancement of NIR-AuNPs by the consideration of the photophysical parameters.

Given that *k*_nr_ depends on the distance and orientation between the fluorophore and AuNPs,^46^ the slight change in *k*_nr_ suggests that the enzyme-triggered aggregation induces a very small disruption of the architecture of the nanostructure, such as the distance between the fluorophore and the AuNPs surface. The *k*_r_ of Lip-Cy5.5m on HGNs drastically increases by a factor of 25, and *k*_nr_ is similar to those of NGal-NIR-AuNPs/CHO-NIR-AuNPs with and without enzyme (Table 1). These results indicate that the enzyme-triggered enhancement of NIR fluorescence results from an aggregation-induced LSPR shift that increases the electromagnetic resonance coupling, which in turn increases *k*_r_, but has little effect on *k*_nr_ (Fig. 4).

## Conclusions

In summary, we developed a novel enzyme-triggered NIR fluorescence enhancement nanosystem, which was obtained by the deliberate design and construction of functionalized AuNPs. The NIR fluorescence on the AuNPs was enhanced by a factor of 7.8 by β-gal. The increase induced by β-gal in electromagnetic resonance coupling between the AuNPs and the fluorophores gave rise to an increase in *k*_r_ and light scattering intensity, both of which substantially contribute to the fluorescence enhancement. This novel strategy for the design of NIR fluorescent sensors will be widely applicable for the development of various enzyme-detection systems by the introduction of specific substrates on AuNPs. The new NIR fluorescence enhancement principle described here will be useful in the in-demand imaging techniques, such as *in vivo* enzyme detection and early diagnostic imaging. Although some challenges remain for the *in vivo* applications, they will be overcome by the integration with some advanced technologies such as stimuli-responsive polymers or active targeting technologies.
