# Supplementary material for: An enzyme-responsive metal-enhanced near-infrared fluorescence sensor based on functionalized gold nanoparticles
Source: Chem Sci. 2015 Jun 19;6(8):4934–9. doi: 10.1039/c5sc01850a (PMC5664366; doi:10.1039/c5sc01850a)
Supplement: Supplementary file 1 [file SC-006-C5SC01850A-s001.pdf]

Electronic Supplementary Material (ESI) for Chemical Science.
This journal is © The Royal Society of Chemistry 2015

Electronic Supplementary Information

An enzyme-responsive metal-enhanced near-infrared fluorescence sensor based on functionalized gold nanoparticles

Zhanghua Zeng,[†] Shin Mizukami,[†,§] Katsumasa Fujita,[‡] and Kazuya Kikuchi*,[†,§]

[†]Division of Advanced Science and Biotechnology, [‡]Department of Applied Physics, Graduate School of Engineering, and [§]Immunology Frontier Research Center, Osaka University, Osaka 565-0871, Japan

kkikuchi@mls.eng.osaka-u.ac.jp

Contents

1. Materials and instruments
2. Synthesis of ligands
3. Experiment section
3. Supporting Tables, Figures, Chart, and Schemes
4. Supporting Reference

1. Materials and instruments

General chemicals and biological samples were purchased from Wako Pure Chemical, Tokyo Chemical Industries (TCI), or Sigma-Aldrich Chemical Company. They were used without further purification, if not mentioned. β-galactosidase (β-gal) from *E. coli* was purchased from Sigma. ESI mass spectra were taken on LCT-Premier XE (Waters). ^{1}H NMR/^{13}C NMR spectra were carried out on JNM-LA400 (JEOL). UV-Vis-NIR absorbance spectra were measured on UV-2450 spectrophotometer (Shimadzu). Fluorescence spectra were carried out on F7000 fluorospectrometer (Hitachi). Particle size distribution and ζ-potential of Dynamic light scattering (DLS) were obtained from nano Partica SZ-100 (Horiba).

2. Synthesis of ligands

Compound 1

To a 20 mL mixture of water and acetone (*v*/*v* = 1/1) containing 4-hydroxy-3-nitrobenzaldehyde (1.67g, 10.0 mmol), a solution of α-D-galactopyranosyl bromide (5.00 g, 12.0 mmol) in acetone 10 mL was slowly added within 5 min at 0°C and stirred for 2 h. Then, it was kept stirring at room temperature (RT) overnight, and filtrated with celite. After washing with acetone several times, it was slowly concentrated to yellowish oil, then was further purified with flash silica gel column chromatography (*n*-hexane/EtOAc = 5/2) to afford colorless solid **1** (2.25 g, 45%).

^{1}H NMR (400 MHz, $CDCl_3$): δ 2.01 (s, 3H), 2.06 (s, 6H), 2.15 (s, 3H), 4.13–4.35 (m, 3H), 5.11 (m, 1H), 5.19 (m, 1H), 5.46 (m, 1H), 5.53 (m, 1H), 7.51 (d, *J* = 8.6 Hz, 1H), 8.16 (d, *J* = 8.5 Hz, 2H), 8.37 (s, 1H); 10.02 (s, 1H, CHO); MS (ESI^{+}): *m/z* 498.1 (calcd for $[M+H]^{+}$: 498.1).

Compound 2

$NaBH_4$ (0.24 g, 6.1 mmol) solid was slowly added portion-wise under ice-water bath to 60 mL of the mixed solution of $CHCl_3$ and *i*PrOH (*v*/*v* = 3/1) containing compound **1** (1.48 g, 3.0 mmol). After

it was slowly reached ambient temperature, it was kept stirring for 4 h. An aqueous solution containing 10% citric acid (*w*/*w*) 150 mL was poured into it to terminate the reaction. After washed with a 10% $NaHCO_3$ solution (*w*/*w*) 50 mL × 3 and water 150 mL, the bottom phase was dried over Na_2SO_4 overnight and evaporated to give yellow solid. It was subjected to purification with flash silica gel chromatography (EtOAc /*n*-hexane = 2/3) to obtain compound **2** as colorless solid (1.0 g, 67%).

^{1}H NMR (400 MHz, $CDCl_3$): δ 2.03 (s, 3H), 2.08 (s, 6H), 2.19 (s, 3H), 4.03 (1H), 4.21 (1H), 4.28 (1H), 4.61 (s, 2H), 5.02 (m, 1H), 5.15 (m, 1H), 5.44 (m, 1H), 5.55 (m, 1H), 7.16 (d, *J* = 8.3 Hz, 1H), 7.46 (d, *J* = 8,4 Hz, 1H), 8.12 (s, 1H); MS (ESI$^+$): *m/z* 500.2 (calcd for $[M+H]^+$: 500.1).

Compound 3

p-Nitrophenyl chloroformate (0.96 g, 4.5 mmol) was dissolved in CH_2Cl_2 2.0 mL and was dropwisely added to a mixture of compound **2** (0.95 g, 2.0 mmol) in pyridine 1.0 mL and CH_2Cl_2 30 mL The mixed solution was stirred at ambient temperature for 20 h and diluted with CH_2Cl_2 60 mL. After washing with 10% citric acid (*w*/*w*) 100 mL and 5% $NaHCO_3$ aqueous solution (*w*/*w*) 100 mL, the bottom phase was dried over Na_2SO_4 overnight and evaporated to give yellow solid. It was subjected to purification with flash silica gel chromatography (EtOAc /*n*-hexane = 1/1) to obtain solid compound **3** (1.0 g, 78%).

^{1}H NMR (400 MHz, $CDCl_3$): δ 1.98 (s, 3H), 2.05 (s, 6H), 2.16 (s, 3H); 4,07–4.15 (m, 1H), 4.21-4.31 (m, 2H), 5.03 (m, 1H), 5.17 (m, 1H), 5.19 (s, 2H, CH_2O), 5.48–5.53 (m, 2H), 7,14 (d, *J* = 8,6 Hz, 1H), 7.39 (d, *J* = 8.8 Hz, 1H), 7.49 (d, *J* = 8.2 Hz, 2H), 8.13 (s, 1H), 8.27 (d, *J* = 8.7 Hz, 2H); MS (ESI$^+$): *m/z* 656.2 (calcd for $[M+H]^+$: 656.1).

Compound 4

To the mixed solution of CH_2Cl_2 30 mL and of 2,2-(ethylenedioxy)bis(ethylamine) 15 mL (100 mmol), di-*tert*-butyl dicarbonate (6.5 g, 30 mmol) in CH_2Cl_2 200 mL was dropwisely added within 1 h under ice-water bath. The mixture was slowly allowed to reach ambient temperature and kept stirring

overnight. It was extracted with brine 200 mL × 3 and water 200 mL. The organic layer was dried over Na_2SO_4 overnight and evaporated to obtain colorless compound mono-*tert*-butoxycarbonylated 2,2-(ethylenedioxy)bis(ethylamine) (3.2 g, 74%).

^{1}H NMR (400 MHz, $CDCl_3$): δ 1.47 (s, 9H), 2.88 (t, *J* = 4 Hz, 2H), 3.28 (d, *J* = 4.2 Hz, 2H), 3.51 (m, 4H), 3.62 (s, 4H), 5.32 (s, br, 1H); MS (ESI$^+$): *m/z* 249.2 (calcd for $[M+H]^+$: 249.2).

Then, to 25 mL of CH_2Cl_2 solution containing lipoic acid (2.06 g, 10.0 mmol). WSCD·HCl (2.4 g, 12.5 mmol) and triethylamine (1.32 g, 13.1 mmol) were slowly added into the solution. After it was stirred at RT for half hour, 5.0 mL of CH_2Cl_2 solution of *N*-boc-2,2-(ethylenedioxy)bis(ethylamine) (2.6 g, 10.5 mmol) was dropwisely added, and the reaction solution was continuing stirring at RT for 2 h. An aqueous 10% (*w*/*w*) citric acid solution was slowly added to quench the reaction, and it was extracted three times with CH_2Cl_2. After dried over Na_2SO_4 overnight, the organic phase was evaporated to give yellowish oil compound **4** (3.6 g, 82%).

^{1}H NMR (400 MHz, $CDCl_3$): δ1.22–1.33 (m, 2H),1.49 (s, 9H), 1.60–1.7 (m, 4H), 1.90 (m, 1H), 2.20 (t, *J* = 6.0 Hz, 2H), 2.46 (m, 1H), 3.10–3.20 (m, 2H), 3.33 (m, 2H), 3.45 (d, 2H), 3.51–3.54 (m, 1H), 3.58 (t, *J* = 4.8 Hz, 4H), 3.69 (s, 4H); MS (ESI$^+$): *m/z* 437.18 (calcd for $[M+H]^+$: 437.21).

Compound 5

Compound **4** (1.73 g, 4.0 mmol) was dissolved in 5 mL of mixed solution of TFA and CH_2Cl_2 (*v*/*v* = 2/3) at 0 °C. This reaction solution slowly reached RT and stirred for 4 h. After the solvent was removed, the residual yellowish oil was redissolved in CH_2Cl_2 2.0 mL. To the solution, succinic anhydride (0.4 g, 4.0 mmol) and triethylamine (1.01 g, 10.0 mmol) were dropwisely added within 10 min, and the mixed reaction solution was kept stirring for 24 h. It was washed with brine 200 mL and water 200 mL, dried over Na_2SO_4, and evaporated to give yellow oil **5** (1.71 g, 84%).

^{1}H NMR (400 MHz, $CDCl_3$): δ 1.28–1.81 (m, 6H), 1.91 (m, 1H), 2.15–2.24 (m, 2H), 2.42-2.52 (m, 5H), 3.01–3.12 (m, 2H), 3.29-3.37 (m, 4H), 3.45–3.62 (m, 9H); MS (ESI$^+$): *m/z* 437.2 (calcd for $[M+H]^+$: 437.2).

Compound 6

Compound **5** (0.87 g, 2.0 mmol), *N*-Boc-2,2-(ethylenedioxy)bis(ethylamine) (0.51 g, 2.0 mmol) and WSCD·HCl (0.80 g, 4.1 mmol) were mixed and dissolved in 25 mL CH_2Cl_2. Triethylamine (0.66 g, 6.6 mmol) in CH_2Cl_2 5.0 mL was dropwisely added into the mixed reaction solution under ice-water bath. It was kept stirring for 3 h at ambient temperature. An aqueous solution containing 10% citric acid (*w*/*w*) was slowly added into it to terminate the reaction. It was extracted three times. The bottom organic layer was combined together and concentrated to give compound **6** (0.85 g, 64 %).

^{1}H NMR (400 MHz, $CDCl_3$): δ 1.22–1.37 (m, 2H), 1.45 (s, 9H), 1.56–1.75 (m, 4H), 1.91 (m, 1H), 2.13–2.23 (m, 2H), 2.44-2.55 (m, 5H), 2.98–3.16 (m, 2H), 3.24–3.36 (m, 8H), 3.45–3.75 (m, 17H); MS (ESI$^+$): *m/z* 667.4 (calcd for $[M+H]^+$: 667.3).

Compound 7

Compound **6** (0.66 g, 1.0 mmol) dissolved in mixed solvent of CH_2Cl_2 and TFA (*v*/*v* = 3/2) 5.0 mL under ice-water bath. The mixed reaction solution was slowly allowed to reach ambient temperature and kept stirring for 4 h. The organic solvent was evaporated under vacuum to afford yellowish oil. This oil was redissolved in mixed solvent of DMF and triethylamine (*v*/*v* = 4/1) 2.0 mL and DMF solution 5.0 mL of compound **3** (0.67 g, 1.0 mmol) was dropwisely added into it within 15 min. The mixed reaction solution was kept stirring overnight at RT. After washing with 10% citric acid 100 mL and 5% $NaHCO_3$ aqueous solution (*w*/*w*) 100 mL, the bottom phase was combined together, dried over Na_2SO_4 overnight and concentrated under vacuum to give yellow residue. It was further separated on flash chromatography (*n*-hexane/EtOAc = 1/1) to obtain compound **7** (0.63 g, 58%) as colorless solid.

^{1}H NMR (400 MHz, $CDCl_3$): δ 1.27–1.39 (m, 2H), 1.61–1.77 (m, 4H), 1.91-1.98 (m, 1H), 2.04 (s, 3H), 2.11 (m, 6H), 2.17 (t, *J* = 6.4 Hz, 2H), 2.22 (s, 3H), 2.44–2.54 (m, 5H), 3.16–3.67 (m, 25H), 5.07 (m, 1H), 5.12 (s, 2H), 5.19 (m, 1H), 5.44 (m, 1H), 5.55 (m, 1H), 7,16 (d, *J* = 8,5 Hz, 1H), 7.36 (d, *J* = 8.9 Hz, 1H), 7.84 (s, 1H); MS (ESI$^+$): *m/z* 1092.4 (calcd for $[M+H]^+$: 1092.4).

Lip-NGal

$NaOCH_3$ (54 mg, 1.0 mmol) was dissolved in CH_3OH 1.0 mL and added slowly to the CH_3OH solution containing compound **7** (0.55 g, 0.50 mmol) under ice-water bath. The mixed reaction solution was kept stirring for 4 h at this temperature. It was neutralized to pH 7 using 1% HCl aq. After filtering off insoluble residue, the filtrate was collected and concentrated. The obtained solid was subjected to purification on flash silica gel column chromatography (CH_2Cl_2/CH_3OH = 10/1) to give compound **Lip-NGal** (0.337 g, 73%) as colorless solid after evaporation of solvent.

^{1}H NMR (400 MHz, $CDCl_3$): δ 1.26–1.48 (m, 2H), 1.61–1.66 (m, 4H), 1.86-1.92 (m, 1H), 2.17-2.21 (m, 2H), 2.42–2.45 (m, 5H), 3.07-3.20 (m, 2H), 3.35–3.58 (m, 25H), 3.71 (m, 1H), 3.80 (s, 2H), 3.93 (m, 1H), 4.02 (m, 1H), 4.51 (m, 1H), 5.02 (s, 2H), 6.36 (s, 1H), 6.81 (s, 1H), 7.07-7.12 (m, 2H), 7.32 (s, 1H), 7.49 (s, 1H), 7.74 (s, 1H); ^{13}C NMR (75 MHz, $CDCl_3$): δ 173.5, 172.8, 156.4, 149.8, 139.9, 134.2, 131.4, 124.8, 118.1, 101.9, 75.5, 73.3, 70.9, 70.1, 69.9, 69.4, 68.8, 64.8, 61.5, 56.5, 40.8, 40.2, 39.3, 39.2, 38.5, 36.2, 34.6, 31.5, 28.9, 25.4; MS (ESI$^+$): *m/z* 924.3743 (calcd for $[M+H]^+$: 924.3582); HRMS (FAB$^+$): *m/z* 924.4 (calcd for $[M+H]^+$: 924.4).

3-Formyl-4-hydroxybenzoic acid

In a 500 mL flask, *p*-hydroxybenzoic acid (3.0 g, 22 mmol) was suspended in trifluoroacetic acid 10 mL. Hexamethylenetetramine (3.1 g, 22 mmol) dissolved in trifluoroacetic acid was dropwisely added under argon atmosphere. After it was heated at reflux under argon atmosphere for 2 h, it turned a transparent yellowish solution. When it cooled to RT, the solution was stirred with 4 M HCl aq. 300 mL for 3 h. The insoluble yellowish powder was collected, washed with brine, water and acetone three times, and concentrated under vacuum overnight to give 3-formyl-4-hydroxybenzoic acid (1.4 g, 41%).

^{1}H NMR (400 MHz, $CDCl_3$): δ 7.36 (m, 1H), 7.79 (s, 2H), 8.36 (d, *J* = 6.4 Hz, 1H), 10.21 (s, 1H); MS (ESI$^+$): *m/z* 167.1 (calcd for $[M+H]^+$: 167.0).

Compound 8

Lipoic acid (2.06 g, 10.0 mmol), WSCD·HCl (2.40 g, 12.5 mmol) and triethylamine 2.0 mL (13 mmol) were dissolved in CH_2Cl_2 25 mL. The mixed solution was stirred for half hour at RT and 2-(2-aminoethoxy)ethanol (1.10 g, 10.5 mmol) in CH_2Cl_2 5.0 mL was dropwisely added into the mixed solution. It was kept stirring for 2 h at RT. An aqueous solution containing 10% citric acid (*w*/*w*) was poured to wash three times. The bottom organic phase was collected, dried over Na_2SO_4 and evaporated solvent under vacuum to obtain yellow oil compound **8** (2.3 g, 77%).

^{1}H NMR (400 MHz, $CDCl_3$): δ 1.61–1.73 (m, 6H), 1.93 (m, 1H), 2.18 (t, 2H, *J* = 6.2 Hz), 2.43 (m, 1H), 2.50–2.55 (m, 2H), 3.33–3.79 (m, 9H); MS (ESI$^+$): *m/z* 294.1 (calcd for $[M+H]^+$: 294.1).

Lip-CHO

Compound **8** (1.5 g, 5.0 mmol) and 3-formyl-4-hydroxybenzoic acid (0.83 g, 5.0 mmol) were mixed and dissolved in 20 mL CH_2Cl_2. DCC (6.0 mmol) in 5.0 mL CH_2Cl_2 was slowly added into the mixed solution within 5.0 min. It was kept stirring at ambient temperature for 6 h. Water 50 mL was poured to quench the reaction. After filtering off the precipitate, the bottom organic layer in filtrate was collected, dried overnight and evaporated solvent to obtain yellow oil. It was subjected to a column chromatography (CH_2Cl_2/MeOH = 20/1) to afford Lip-CHO (0.11 g, 5%).

^{1}H NMR (400 MHz, $CDCl_3$): δ 1.25–1.71 (m, 6H), 1.90 (m, 1H), 2.17 (t, 2H), 2.46 (m, 1H), 3.14 (m, 2H), 3.44–3.62 (m, 5H), 3.81 (t, *J* = 5.6 Hz, 2H), 4.49 (t, *J* = 5.6 Hz, 2H), 7.06 (d, *J* = 6.4 Hz, 1H), 8.19 (d, *J* = 6.4 Hz, 1H), 8.34 (s, 2H), 9.97 (s, 1H), 11.43 (s, 1H); ^{13}C NMR (75 MHz, $CDCl_3$): δ 196.3, 172.7, 165.2, 165.1, 137.8, 136.2, 122.1, 120.1, 118.1, 69.9, 69.0, 64.0, 56.4, 40.3, 39.1, 38.4, 36.4, 28.9, 25.3; MS (ESI$^+$): *m/z* 442.1726 (calcd for $[M+H]^+$: 442.1358); HRMS-FAB: *m*/*z* 442.1 (calcd for $[M+H]^+$: 442.1).

Compound 9

Di-*tert*-butyl dicarbonate (6.5 g, 30 mmol) dissolved in 200 mL CH_2Cl_2 was dropwisely added to

CH_2Cl_2 30 mL containing 4,7,10-trioxa-1,13-tridecanediamine 22.0 mL (100 mmol) within 1 h under ice-water bath. The mixed solution was allowed to reached RT and continued stirring overnight. It was extracted with 200 mL brine and 200 mL water three times, respectively. The bottom organic phase was combined together, dried over Na_2SO_4 and evaporated under vacuum to afford colorless oil *N*-Boc-4,7,10-trioxa-1,13-tridecanediamine (6.7 g, 70%).

Lipoic acid (2.06 g, 10.0 mmol), WSCD·HCl (2.40 g, 12.5 mmol), and triethylamine (2.0 mL, 13.2 mmol) were dissolved in CH_2Cl_2 25 mL. After the mixed solution was stirred for half hour at RT, compound *N*-Boc-4,7,10-trioxa-1,13-tridecanediamine (3.3 g, 10.5 mmol) dissolved in CH_2Cl_2 5.0 mL was slowly added to it. The mixture reaction solution was kept stirring for 2 h at RT. An aqueous solution containing 10% citric acid (*w*/*w*) was poured to wash three times. The bottom organic phase was collected, dried over Na_2SO_4 and evaporated under vacuum to give compound **9** (3.8 g, 75%).

^{1}H NMR (400 MHz, $CDCl_3$): δ 1.21–1.33 (m, 2H), 1.39 (s, 9H), 1.56–1.74 (m, 10H), 2.01 (m, 1H), 2.18 (m, 2H), 2.52 (t, 1H), 2.86-3.51 (m, 17H); MS (ESI$^+$): *m/z* 509.3 (calcd for $[M+H]^+$: 509.3).

Compound 10

Compound **9** (2.0 g, 4.0 mmol) was slowly dissolved in TFA and CH_2Cl_2 (*v*/*v* = 2/3) 5.0 mL under ice-water bath. The reaction solution was allowed to slowly reach ambient temperature and kept stirring for 4 h. The organic solvent was evaporated under vacuum to afford yellowish oil. This oil was redissolved in CH_2Cl_2 2.0 mL, subsequently succinic anhydride (0.40 g, 4.0 mmol) and triethylamine (10.0 mmol) were slowly added. The mixed reaction solution was kept stirring overnight, and then it was extracted with 200 mL brine and 200 mL water three times. The bottom organic phase was collected, dried over Na_2SO_4, and then evaporated to obtain compound **10** (1.61 g, 80%).

^{1}H NMR (400 MHz, $CDCl_3$): δ 1.26–1.73 (m, 12H), 2.02 (m, 1H), 2.21 (m, 2H), 2.48–2.61 (m, 5H), 2.85–3.61 (m, 17H); MS (ESI$^+$): *m/z* 509.3 (calcd for $[M+H]^+$: 509.2).

Compound 11

Compound **10** (1.0 g, 2.0 mmol) and *N*-Boc-4,7,10-trioxa-1,13-tridecanediamine (0.65 g, 2.0 mmol) were dissolved in CH_2Cl_2 25 mL. A CH_2Cl_2 solution 5.0 mL of WSCD·HCl (0.80 g, 4.1 mmol) and triethylamine (0.66 g, 6.6 mmol) was slowly added to it. The mixed reaction solution was kept stirring at RT for 3 h. An aqueous solution containing 10% citric acid (*w*/*w*) 150 mL was poured into it to wash three times. The bottom organic phase was combined together, dried over Na_2SO_4 for 12 h, and then concentrated to obtain yellowish oil. It was separated on silica gel column chromatography to give compound **11** (1.1 g, 70%).

^{1}H NMR (400 MHz, $CDCl_3$): δ 1.23–1.32 (m, 2H), 1.38 (s, 9H), 1.45–1.75 (m, 12H), 2.02 (m, 1H), 2.22 (m, 2H), 2.44–2.65 (m, 5H), 2.89–3.59 (m, 35H); ESI-MS: *m/z* 811.5 (calcd for $[M+H]^+$: 811.5).

Compound 12

Compound **11** (0.81 g, 1.0 mmol) and *N*-Boc-11-aminoundecanoic acid (0.30 g, 1.0 mmol) were mixed and dissolved in CH_2Cl_2 15 mL. A CH_2Cl_2 solution 5.0 mL of WSCD·HCl (0.40 g, 2.1 mmol) and triethylamine (0.33 g, 3.3 mmol) was slowly added to it. The mixed solution was stirred for 2 h at RT. An aqueous solution containing 10% citric acid (*w*/*w*) 150 mL was poured to wash three times. The bottom organic phase was combined together, dried over Na_2SO_4 overnight, and then concentrated to oil. The oil was further purified on flash silica gel column chromatography to obtain compound **12** (0.70 g, 71 %).

^{1}H NMR (400 MHz, $CDCl_3$): δ 1.24–1.95 (m, 40H), 2.21–2.27 (m, 4H), 2.42–2.65 (m, 5H), 2.88–3.63 (m, 37H); MS (ESI^+): *m/z* 994.6 (calcd for $[M+H]^+$: 994.6).

Compound 13

Compound **12** (0.5 g, 0.5 mmol) was dissolved in mixed solvent of TFA and CH_2Cl_2 (*v*/*v* = 2/3) 5.0 mL at 0 °C. It was allowed to reach RT and kept stirring for 4 h. The organic solvent was evaporated under vacuum to afford yellowish oil. This oil was redissolved in CH_2Cl_2 5.0 mL, and *N*-Boc-11-aminoundecanoic acid (0.15 g, 0.50 mmol) dissolved in CH_2Cl_2 was slowly added to it. Subsequently,

WSCD·HCl (0.20 g, 1.1 mmol) and a little excess triethylamine 0.25 mL (1.7 mmol) were slowly added to the mixed solution. It was kept stirring at RT for 6 h, and water 10 mL quenched the reaction. It was washed by aqueous solution of 10% citric acid and water three times, and dried over Na_2SO_4. The obtained yellowish oil after concentration of solvent was separated on silica gel column chromatography to afford compound **13** (0.37 g, 61 %).

^{1}H NMR (400 MHz, $CDCl_3$): δ 1.22–1.98 (m, 56H), 2.19–2.26 (m, 6H), 2.42–2.65 (m, 5H), 2.89–3.69 (m, 39H); MS (ESI$^+$): *m/z* 1177.7 (calcd for $[M+H]^+$: 1177.8).

Compound Lip-Cy5.5m

Compound **13** (0.24 g, 0.20 mmol) was dissolved in mixture of CH_2Cl_2 and TFA (*v*/*v* = 3/2) 5.0 mL at 0 °C. It was allowed to reach RT and stirred for 4 h. The organic solvent was evaporated under vacuum to afford yellowish oil. Subsequently, it was redissolved in CH_2Cl_2 5.0 mL, and neutralized by a little excess triethylamine. Cy5.5m (Scheme S4) obtained from published procedure[S1] was dissolved in CH_2Cl_2 5.0 mL, and WSCD·HCl were slowly added to the mixed solution. It was kept stirring at RT for 24 h. 30.0 mL water was slowly added to quench the reaction, and it was extracted three rounds with CH_2Cl_2 50 mL and water 50 mL. The bottom organic phase was combined and dried with Na_2SO_4. Purification was carried on a column chromatography with CH_2Cl_2/MeOH (from 20/1 to 6/1) as eluent to give Lip-Cy5.5m (0.056 g, 17%).

^{1}H NMR (400 MHz, DMSO): δ 1.09–1.99 (m, 65H), 2.18 (s, 8H), 2.42 (m, 5H), 2.89– 3.08 (m, 14H), 3.1-3.41 (m, 25H), 3.65 (s, 3H), 4.15 (br, 2H), 6.23 (m, 4H), 6.58 (m, 1H), 7.43 (m, 2H), 7.60-7.75 (m, 10H), 8.11–8.19 (m, 4H), 8.19 (d, *J* = 8.0 Hz, 2H), 8.38 (m, 2H); MS (ESI$^+$): *m/z* 1642.8 (calcd for M^+: 1642.0); HRMS (FAB$^+$): *m/z* 1642.0404 (calcd for M^+: 1642.0418).

3. Experiment section

Synthesis of functionalized gold nanoparticles

Citrate-capped gold nanoparticles. Citrate-coated gold nanoparticles was obtained by the previous

methods. Thirty milliliter aqueous solution of 2.0 mM of $HAuCl_4$ hydrate was allowed to heat at reflux for 30 min, and aqueous solution of 75 mM trisodium citrate 3.5 mL was quickly added into it. The change of reaction solution color occurred immediately from yellow, to purple and finally wine red. The mixed reaction solution was kept boiling for 30.0 min, and then cooled slowly to RT.

NGal-NIR-AuNPs/CHO-NIR-AuNPs. Ten milliliter solution of citrate-coated gold nanoparticles obtained above was centrifuged three rounds by 10,000 rpm. The pellet collected was re-dispersed in PBS (pH 7.4, 50.0 mM, 10.0 mM KCl, 10.0 mM $MgCl_2$). Then, PBS (containing 2% DMSO) 5.0 mL containing 0.1 mM Lip-NGal (Lip-CHO), PBS (containing 2% DMSO) 5 mL containing 0.02 mM Lip-Cy5.5m and PBS 5 mL containing 0.1 mM Lip-PEG_{400} were fully mixed and quickly dropped into 5.0 mL solution of citrate-coated gold nanoparticles. The mixed solution was incubated and shaken under the dark at RT for 6 h. After filtering off the insoluble residue, the collected filtrate was centrifuged several rounds by 10,000 rpm, until no Lip-Cy5.5m was observed in supernatant. The pellet was collected and redispersed in PBS to give corresponding NGal-NIR-AuNPs and CHO-NIR-AuNPs, respectively. NGal-NIR-AuNPs/CHO-NIR-AuNPs was obtained by mixing the equivalent volume of NGal-NIR-AuNPs and CHO-NIR-AuNPs.

NGal-AuNPs/CHO-AuNPs. NGal-AuNPs and CHO-AuNPs were prepared by same method as NGal-NIR-AuNPs and CHO-NIR-AuNPs, in which Lip-Cy5.5m was not used. NGal-AuNPs/CHO-AuNPs was obtained by mixing the equivalent volume of NGal-AuNPs and CHO-AuNPs.

Hollow gold nanoparticles (HGNs). HGNs were prepared following the reported method.[32] Briefly, silver nitrate 0.04 g and poly(vinylpyrrolidone) (PVP, Mw ~30,000, 1.0 g) were mixture and suspended in ethylene glycol 20 mL. The mixed suspension was allowed to heat at 160 °C for 2 h under dark, and then cooled slowly to RT. The silver nanoparticles (AgNPs) obtained using this method showed very stable monodispersions in aqueous solution without addition of stabilizing agents. 1.0 mL of AgNPs prepared was diluted in DI water 25.0 mL and then heated to reflux for 10 min before a 1.5 mL of 2.0 mM aqueous $HAuCl_4$ was dropwisely added. A sequential color variation from yellow to dark blue occurred during the time of the Au-Ag replacement reaction. The mixed solution was slowly cooled to

RT, after 20 min of stirring. It was purified three rounds by centrifugation at 5,000–7,000 rpm. The collected pellet was redispersed in 5.0 mL solution of 0.3 mM trisodium citrate and stored at 4°C.

Lip-Cy5.5m on HGNs. To a 1.0 mL solution of obtained citrate-coated gold nanoparticles, 2.0 mL of 0.02 mM Lip-Cy5.5m in PBS (containing 2% DMSO) and 2.0 mL of 0.1 mM Lip-PEG_{400} in PBS were quickly mixed and added dropwisely. The mixture solution was incubated at room temperature overnight. After filtering off the insoluble residue, the collected filtrate was centrifuged three rounds by 5,000 rpm, until there was no Lip-Cy5.5m in supernatant by checking with an absorbance spectrophotometer. The pellet was redispersed in specific volume of PBS to give desired concentration of Lip-Cy5.5m on hollow gold nanoparticles.

HPLC Analysis. Preparative HPLC was performed with an Inertsil ODS-3 (10.0 mm × 250 mm) column (GL Sciences Inc.) using an HPLC system that comprised a pump (PU-2087, JASCO) and a detector (UV-2075, JASCO).

Quantitative determination of ligands amount on NGal-NIR-AuNPs/CHO-NIR-AuNPs. In the preparation of NGal-NIR-AuNPs, all of the supernatant was collected and subjected to measurement by UV-Vis-NIR spectrophotometer. The amount of Lip-Cy5.5m (C4) on AuNPs was determined by amount of Lip-Cy5.5m (A4) in original addition and surplus (B4) in supernatant after conjugation to AuNPs, according to the molar extinction coefficient of Lip-Cy5.5m. After that, the collected supernatant was lyophilized to give blue solid. It was dissolved by sonication in the mixture of H_2O/acetonitrile (1:1), and the suspended Lip-Cy5.5m as pellet was collected by centrifugation at 4,000 rpm. It was redissolved in the mixture of H_2O/acetonitrile (1:1) and centrifuged. This process was repeated by several times, and then the supernatants were lyophilized to afford yellow solid. Finally, it was dissolved in specific volume of the mixture of H_2O/acetonitrile (1:1), and subjected to quantitative analysis by HPLC. The amount of Lip-NGal (C1) and Lip-PEG_{400} (C3) on AuNPs was determined by amount of Lip-Cy5.5m (A1) and Lip-PEG_{400} (A3) in original addition and surplus (B1, B3) in supernatant after conjugation to AuNPs, according to HPLC determination (Scheme S4).

The amount of Lip-CHO (C2), Lip-PEG_{400} (C3) and Lip-Cy5.5m (C4) on CHO-NIR-AuNPs was obtained by the same method as above.

Fluorescence quantum yield. The samples were dissolved in PBS to obtain desired concentrations. The relative fluorescence quantum yield of fluorophore in each sample was estimated in 50 mM PBS buffer (pH 7.4) using 3,3-diethylthiadicarbocyanine iodide in EtOH (Φ = 0.35) as standard reference for comparison. To estimate molar extinction coefficient of Lip-Cy5.5 and fluorescence quantum yield on functionalized gold nanoparticles without and with enzyme, the dye-free gold nanoparticles NGal-AuNPs/CHO-AuNPs was prepared following same procedure as NGal-NIR-AuNPs/CHO-NIR-AuNPs. We assumed that the Lip-Cy5.5 has negligible effect on the enzymatic reaction, Schiff formation and subsequent aggregation of gold nanopartices, since the ratio of Lip-Cy5.5 is very small. The molar extinction coefficients of Lip-Cy5.5 without and with enzyme at 6 h were roughly determined according to absorbance differential of NGal-AuNPs/CHO-AuNPs and NGal-NIR-AuNPs/CHO-NIR-AuNPs, and the fluorescence quantum yield could be obtained in term of equation S1 below:

$$\Phi_F = \Phi_R \frac{I_F}{I_R} \frac{\varepsilon_F}{\varepsilon_R} \frac{n_F^2}{n_R^2} \quad \textbf{eq. S1}$$

, where Φ is the quantum yield, I is the integrated intensity, n is the refractive index, and ε is the molar extinction coefficient. The subscript R and F refers to the reference fluorophore (3,3-diethylthiadicarbocyanine iodide) and Lip-Cy5.5, respectively.

TEM measurement. TEM images were recorded at an accelerating voltage of 200 kV using a Hitachi HF-2000. The samples of NGal-NIR-AuNPs/CHO-NIR-AuNPs (2.0 nM) in the absence and presence of enzyme (6 h) and Lip-Cy5.5m on hollow gold nanoparticles were prepared at 37 °C. These samples were deposited onto a carbon film supported on a copper grid by a drop of a PBS dispersion of nanoparticles. The excess solution was removed by an adsorbent paper, and the samples were further dried on vacuum at room temperature.

Light scattering measurement. The light scattering intensity was carried out on Hitachi F7000 fluorospectrometer. Samples of NGal-AuNPs/CHO–AuNPs (1.0 nM) for light scattering was suspended in 50.0 mM PBS (pH 7.4). The excitation light scattering was collected from 400 to 860 nm in the absence and presence of enzyme (6.0 h). The light scattering intensity was extracted and plotted versus wavelength to afford light scattering spectra.

Fluorescence lifetime measurement and decay rate parameters. The fluorescence decay of the sample was estimated by the life time measurement in 50 mM PBS buffer (pH 7.4) with λ_{ex} = 600 nm and λ_{em} = 705 nm. Data analysis was performed with IBH DAS6 software (HORIBA) and the goodness of fit judged in terms of a χ^2 value. The decay time data were analyzed using a sum exponential, employing a nonlinear least-squares re-convolution analysis. The sum of the pre-exponential components (α_i), $\Sigma\alpha_i$, was given normalized to unity. Average fluorescence lifetimes (τ_{ave}) were calculated as $\Sigma\alpha_i\tau_i$.. The radiative (k_r) and nonradiative (k_{nr}) decay rate constants are calculated according to the equations S2 and S3.

$$k_r = \Phi_F / \tau_{ave} \qquad \text{eq. S2}$$
$$k_{nr} = 1/\tau_{ave} - k_r \qquad \text{eq. S3}$$

4. Supporting Tables, Figures, Chart, and Schemes

Table S1. Photophysical properties of Cy5.5m and Lip-Cy5.5m in PBS.

	$\lambda_{abs,max}$ (nm)	$\lambda_{em,max}$ (nm)	ε (M^{-1} cm^{-1})	Φ_F
Cy5.5m	679	705	174,000	0.22
Lip-Cy5.5m	678	707	171,000	0.18

Table S2. Fluorescence lifetime analysis of Lip-Cy5.5m with nanoparticles by time-correlated single-photon counting.

Samples	τ_1 (ps)	τ_2 (ps)	α_1	α_2	τ_{ave} (ps)	χ^2
Lip-Cy5.5m	–	–	–	–	835	1.53

NGal-NIR-AuNPs/CHO-NIR-AuNPs without enzyme	94	701	0.70	0.30	274	1.24
NGal-NIR-AuNPs/CHO-NIR-AuNPs with enzyme (6 h)	118	611	0.82	0.18	202	1.47
Lip-Cy5.5m@HGNs	82	601	0.93	0.07	118	1.19

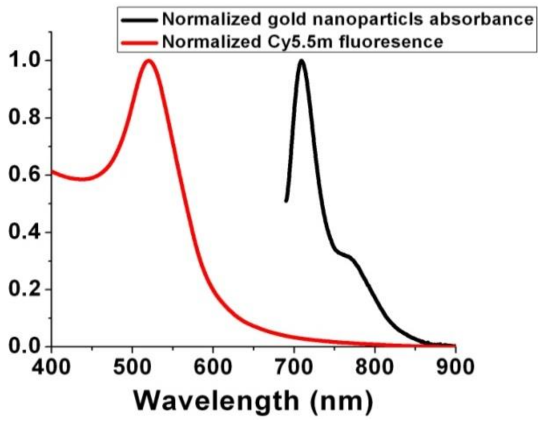

Figure S1. Normalized SPR absorption spectra of gold nanoparticles (red) and fluorescence spectrum of Cy5.5m (black) in PBS (pH 7.4).

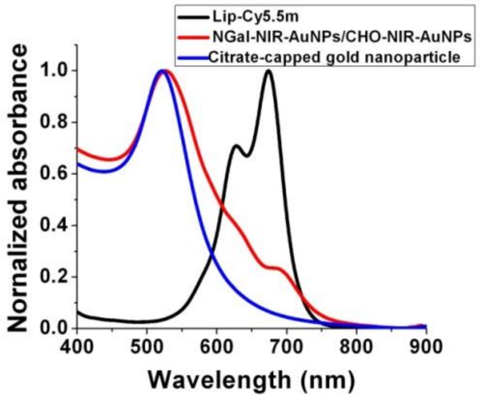

Figure S2. Normalized absorbance spectra of citrate-capped gold nanoparticles (blue), NGal-NIR-AuNPs/CHO-NIR-AuNPs (red), and Lip-Cy5.5m (black) in PBS (pH 7.4).

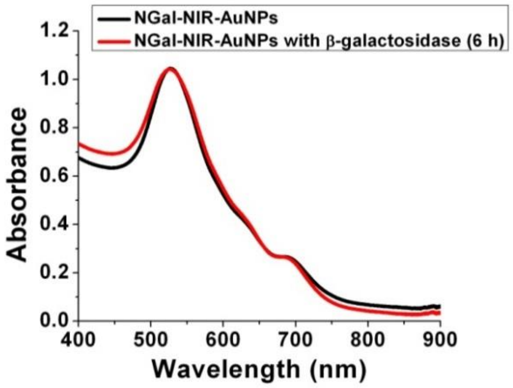

Figure S3. Absorbance spectral variation of NGal-NIR-AuNPs (2.0 nM) with β-gal (1.0 μM) in PBS (pH 7.4) at 37 °C.

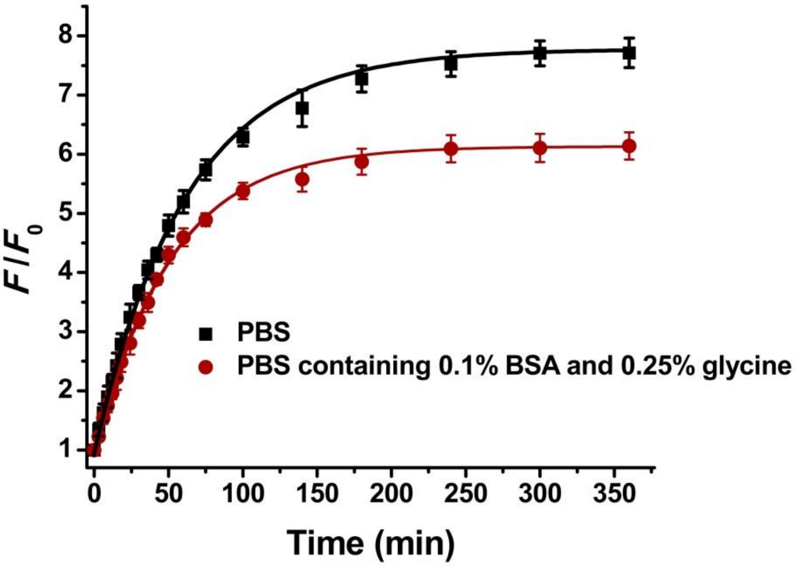

Figure S4. Time-dependent fluorescence enhancement (F/F_0) of NGal-NIR-AuNPs /CHO-NIR-AuNPs (2.0 nM) in the presence of β-gal. Where F referred to the fluorescence intensity in various incubation time, and F_0 referred to the fluorescence intensity in the absence of β-gal.

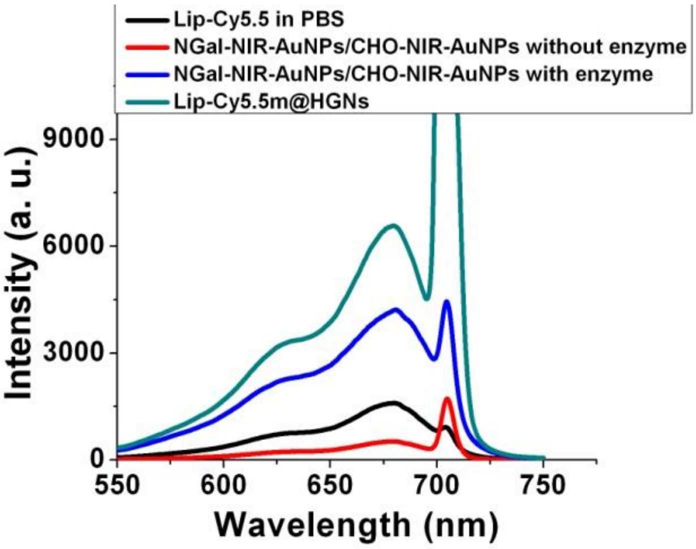

Figure S5. Excitation spectra (λ_{em} = 707 nm) of Lip-Cy5.5m, NGal-NIR-AuNPs/CHO-NIR-AuNPs without and with enzyme (6 h incubation), and Lip-Cy5.5m on hollow gold nanoparticles in PBS (pH 7.4).

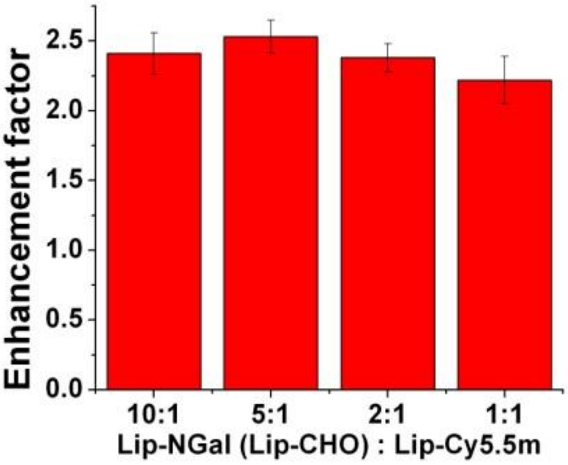

Figure S6. Relative fluorescence intensity of NGal-NIR-AuNPs/CHO-NIR-AuNPs (2.0 nM) with various ratios of Lip-NGal (Lip-CHO) and Lip-Cy5.5m in the presence of β-gal. The ratio values represent the concentration ratio in the modification reaction, and the enhancement factor of Lip-Cy5.5m was normalized as 1. All of samples were measured in PBS (pH 7.4) at 37 °C.

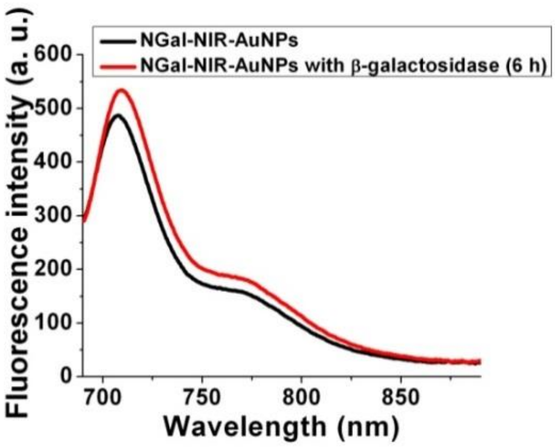

Figure S7. Emission spectra (λ_{ex} = 680 nm) of NGal-NIR-AuNPs (2.0 nM) in the absence and presence of β-gal (1.0 μM). All of samples were measured in PBS (pH 7.4) at 37 °C.

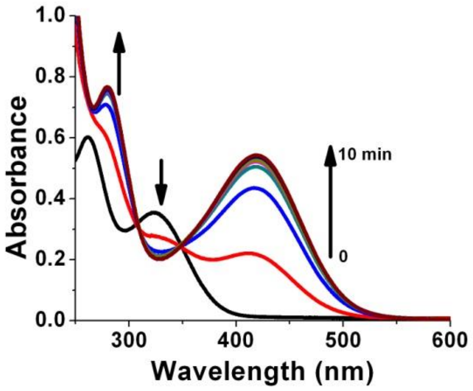

Figure S8. Time-dependent (interval: 1 min) absorbance spectral changes of Lip-NGal (100 μM) in the presence of β-gal (1.0 μM) in PBS (pH 7.4) at 37 °C.

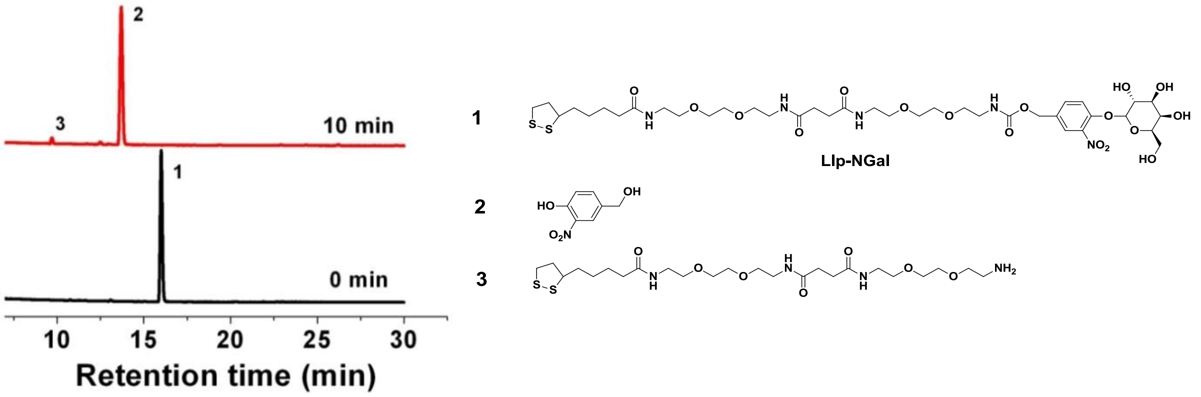

Figure S9. RP-HPLC analysis of enzymatic reaction of Lip-NGal (100 μM) with β-gal (1.0 μM) in PBS (pH 7.4) at 37 °C. Detection wavelength: 260 nm.

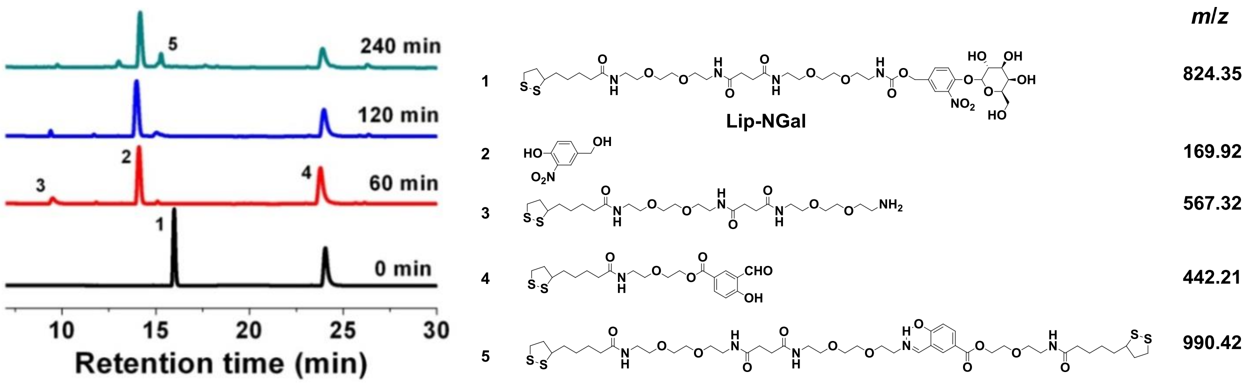

Figure S10. RP-HPLC analysis of Schiff base formation reaction of Lip-NGal (100 μM) and Lip-CHO (100 μM) with β-gal (1.0 μM) in PBS (pH 7.4) at 37 °C. Detection wavelength: 260 nm.

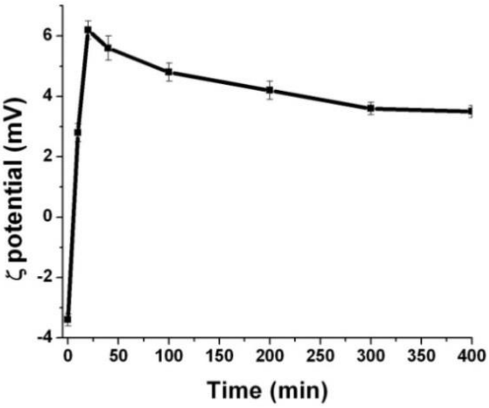

Figure S11. Time-dependent variation of ζ-potential of NGal-NIR-AuNPs/CHO-NIR-AuNPs (2.0 nM) after addition of β-gal (1.0 μM) in PBS (pH 7.4) at 37 °C.

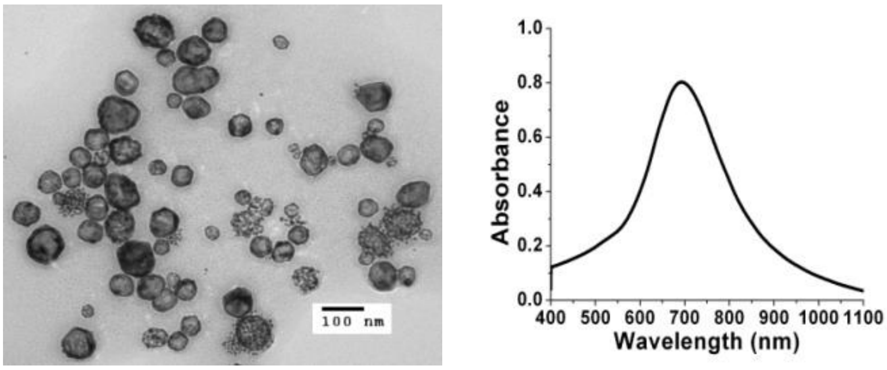

Figure S12. TEM image (left) and absorbance spectrum (right) of HGNs modified with Lip-Cy5.5m, showing its SPR peak at 700 nm. Scale bar: 100.0 nm.

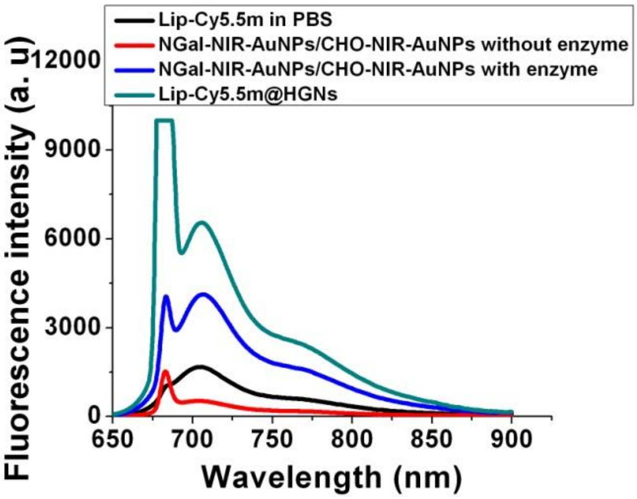

Figure S13. Emission spectra (λ_{ex} = 680 nm) of Lip-Cy5.5m, NGal-NIR-AuNPs/CHO-NIR-AuNPs without enzyme, with enzyme (6 h), and Lip-Cy5.5m on HGNs in buffer.

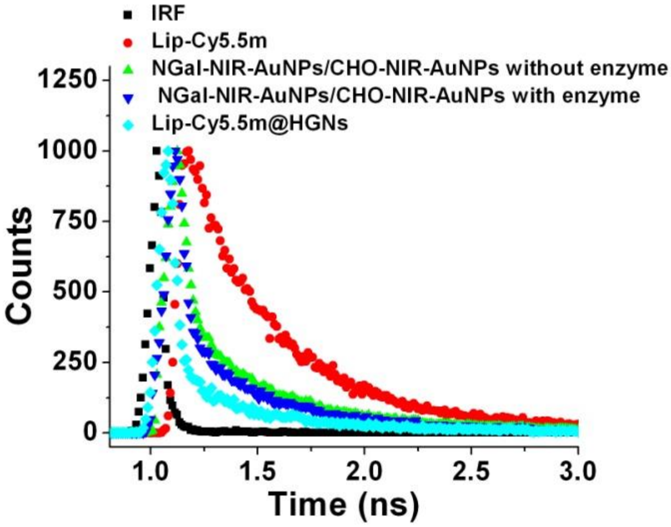

Figure S14. Time-resolved fluorescence decay analysis (λ_{ex} = 600 nm, λ_{em} = 705 nm) in PBS (pH 7.4) at 37 °C. IRF: instrument response function.

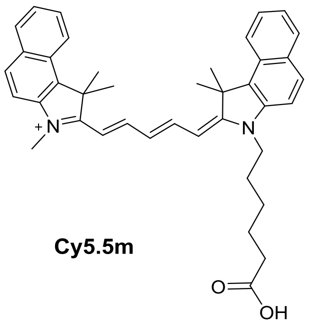

Chart S1. The structure of Cy5.5m

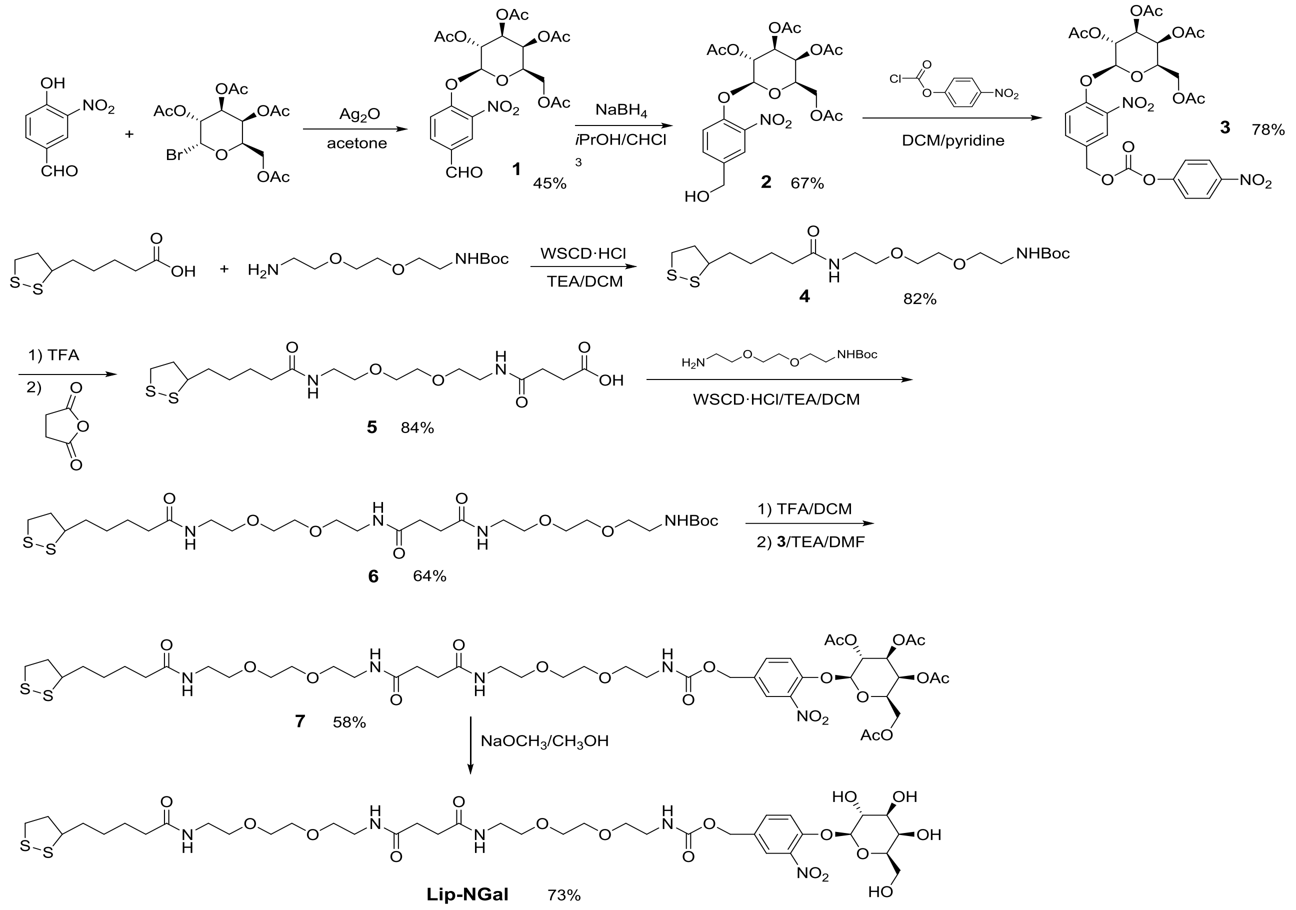

Scheme S1. The synthetic route to Lip-NGal

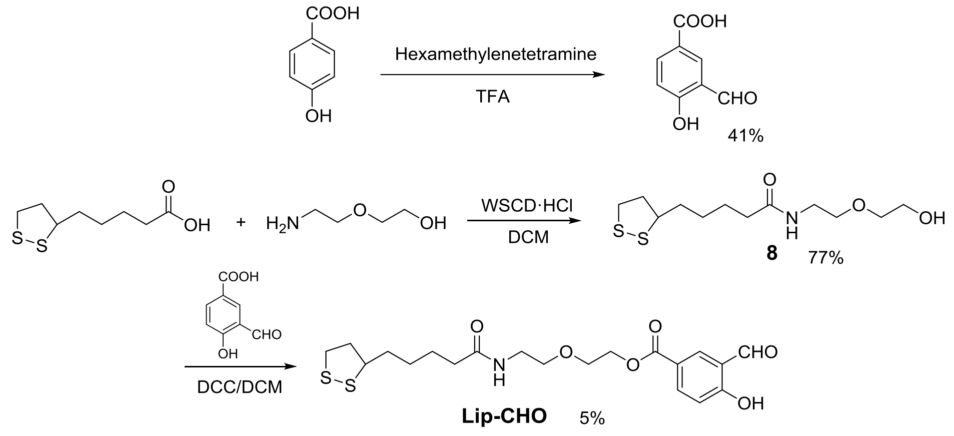

Scheme S2. The synthetic route to Lip-CHO

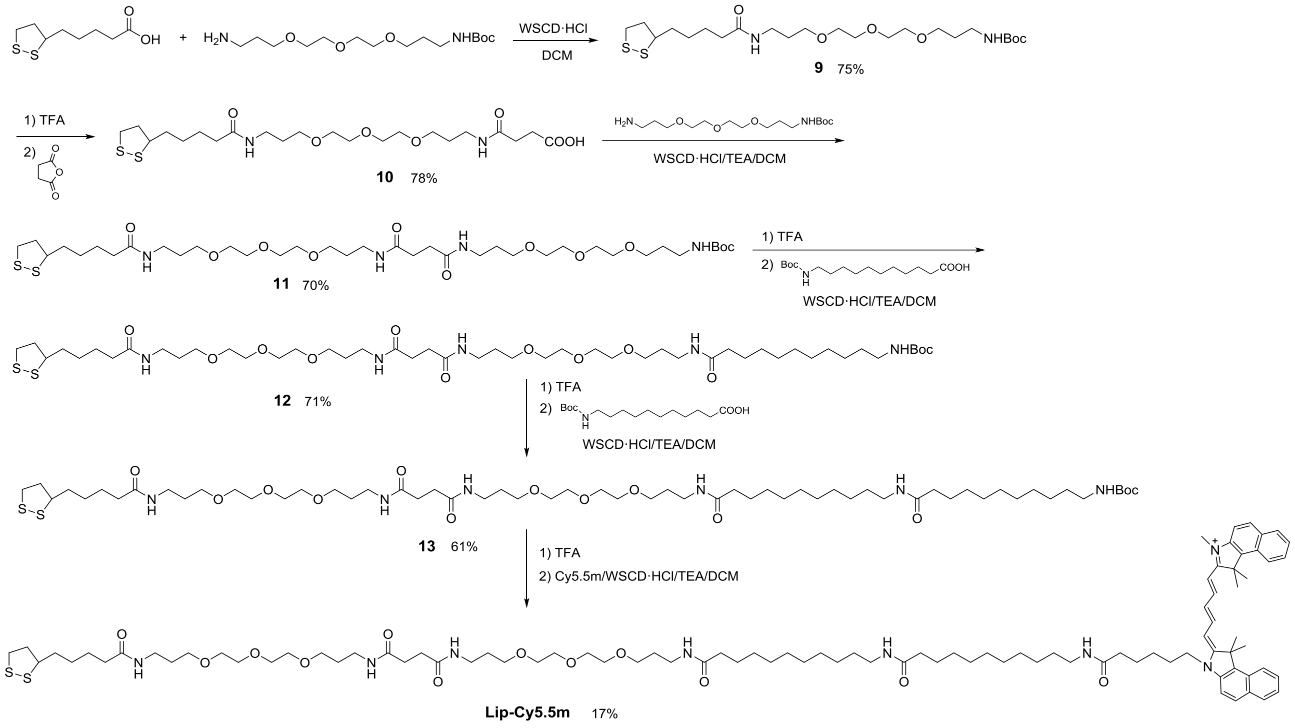

Scheme S3. The synthetic route to Lip-Cy5.5m

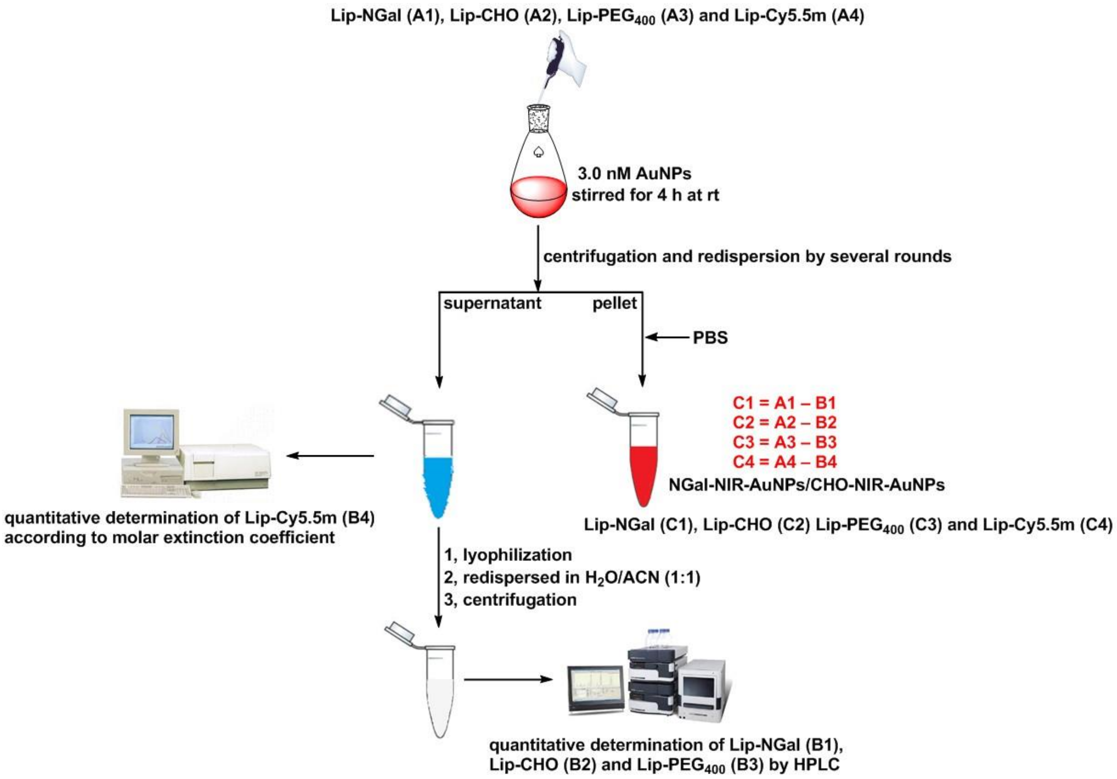

Scheme S4. Schematic illustration of amount quantification of Lip-NGal (C1), Lip-CHO (C2), Lip-PEG_{400} (C3) and Lip-Cy5.5m (C4) on AuNPs.

5. Supporting Reference

S1. M. V. Kvach, A. V. Ustinov, I. A. Stepanova, A. D. Malakhov, M. V. Skorobogatyi, V. V. Shmanai, and V. A. Korshun, *Eur. J. Org. Chem.*, 2008, **12**, 2107–2117.
